# PAIRED ASSOCIATIVE STIMULATION IMPROVES MOTOR FUNCTION IN THE UPPER EXTREMITY IN CHRONIC INCOMPLETE SPINAL CORD INJURY: A CORROBORATIVE STUDY

**DOI:** 10.2340/jrm.v56.41021

**Published:** 2024-11-13

**Authors:** Carl WAHLGREN, Richard LEVI, Adjunct PROFESSOR, Magnus THORDSTEIN

**Affiliations:** 1 Department of Rehabilitation Medicine, Linköping University Hospital, Linköping; 2 Department of Health, Medicine and Caring Sciences, Linköping University, Linköping; 3 Department of Clinical Neurophysiology, Linköping University Hospital, Linköping; 4 Department of Biomedical and Clinical Sciences, Division of Neurobiology, Linköping University, Sweden

**Keywords:** electrical stimulation therapy, rehabilitation, spinal cord injuries, therapy, transcranial magnetic stimulation

## Abstract

**Objective:**

To corroborate findings suggesting that spinally targeted paired associative stimulation improves upper extremity motor function in chronic incomplete spinal cord injury.

**Design:**

Prospective interventional study.

**Subjects:**

Five adults with chronic tetraplegia.

**Methods:**

Participants received paired associative stimulation, combining peripheral nerve stimulation and navigated transcranial magnetic stimulation towards 1 arm (16 1-h sessions during 4 consecutive weeks, targeting the 3 large nerves). Manual muscle testing (MMT) was performed in 23 muscles in each arm, at 3 time points (pre-stimulation, t_0_; the week following the stimulation period, t_1_; and 4–5 weeks post-stimulation, t_2_). Additionally, grip strength and changes in the Canadian Occupational Performance Measure were assessed.

**Results:**

The mean improvement in manual muscle testing scores in the targeted extremity was +0.49 at t_1_ (*p* = 0.078) and +0.55 at t_2_ (*p* = 0.062). Grip strength in the stimulated extremity increased by 3.2 kg at t_1_ and 3.4 kg at t_2_, and in the non-targeted extremity by 2.2 and 3.6 kg, respectively. Performance and satisfaction increased by 2.1/2.4 points at t_1_, and by 2.0/1.9 points at t_2_.

**Conclusion:**

Paired associative stimulation improved motor function: at the group level, MMT of the stimulated hand (*p* = 0.06) and non-stimulated hand (*p* = 0.04). Most participants achieved clinically relevant improvement. Thus, the results corroborate prior studies. The method may complement conventional rehabilitation for improving upper extremity function in incomplete tetraplegia.

## LAY ABSTRACT

Research has indicated that a particular type of nerve stimulation, so-called spinally targeted paired associative stimulation, can improve hand and arm function in persons with chronic spinal cord injury. In this study, 5 adults with weakness in their hands and arms due to spinal cord injury received 4 weeks of near daily “paired stimulation”. This consisted of combining electrical stimulation of the nerves in the arm, and magnetic stimulation of the brain which targeted the part of the brain responsible for controlling that arm. After stimulation, muscle strength and grip strength increased to a similar extent in both extremities. Performance and satisfaction related to individualized functional goals also improved. Although improvements in this small study did not reach statistical significance, results corroborate previous studies suggesting that this type of stimulation may be a useful clinical adjunct to conventional rehabilitation in persons with muscle weakness after spinal cord injury.

Spinal cord injury (SCI) in the present context entails focal damage to the spinal cord, causing sensorimotor deficits at and below the neurological level of injury (NLI). Autonomic dysfunction also typically occurs, e.g., impairment of bladder, bowel, and sexual functions, as well as a propensity for autonomic dysreflexia in lesions at NLI T6 or rostrally (1). Collectively, these impairments often lead to lifelong disability, decreased quality of life, and major costs at an individual and societal level.

Historically, traumatic SCI typically occurred in predominantly younger persons due to high-energy trauma, such as motor vehicle accidents, gunshot wounds, or sporting accidents (2). In recent decades, however, demographics have changed, with an increasing proportion of low-energy trauma sustained among elderly individuals in falls in the presence of degenerative spinal stenosis. SCI may also be due to non-traumatic aetiologies, e.g., being of vascular and infectious origins (3).

Despite decades of research, to date no “cure” is available for reversing the sensorimotor impairments of SCI. Multimodal neurological rehabilitation using conventional methods remains the current “best practice” for achieving some improvement in function (4, 5). This is achieved primarily by training of spared musculature, development of compensatory skills, and prescription of technical aids, as well as by provision of personal assistance and other societal services.

To address the primary neurological deficits, restorative and reparative approaches have been explored in pre-clinical models, including stem cell transplantations, peripheral nerve grafts, antibody therapies, and combinations thereof (6–11). Sophisticated attempts at translating such therapies to clinical practice have encountered several challenges, including lack of efficacy of functional restoration, adverse immune reactions, infections, and development of neuropathic pain (6–11).

As the concept of neuroplasticity in the context of influencing neurological function has gained acceptance (12, 13), alternative research approaches using electrical stimulation of various kinds, designated “neuromodulation”, have increasingly been employed. Such stimulation has been performed invasively (i.e., using implanted electrodes placed epidurally (14) or deep within the brain), as well as non-invasively, using transcutaneous electrical stimulation, which, when targeting the brain, have included either transcranial direct current stimulation (tDCS), transcranial alternating current stimulation, transcranial random noise stimulation or, when targeting the spinal cord, transspinal direct current stimulation (tsDCS, (15–17)). Peripheral nerve stimulation (PNS) has also been tried (18).

In addition, noninvasive neuromodulation using electromagnetic coils, transcranial magnetic stimulation (TMS) targeting neurons in the brain (19, 20), electromagnetic stimulation of the spinal cord (21), or peripheral nerves (22), all representing alternatives or complements to direct electrical stimulation, have also been suggested.

One of the most promising neuromodulatory approaches for clinical implementation combines repetitive TMS (rTMS) with repetitive PNS (rPNS). Studies from Finland (23–25) and the United States (26) have employed protocols targeting the corticospinal synapse with convergent descending rTMS-induced volleys and simultaneous ascending rPNS induced signals from electrical stimulation of peripheral nerves in the targeted extremity, so-called paired associative stimulation (PAS) or paired corticospinal–motoneuronal stimulation. Briefly, the method builds upon the principle of spike timing-dependent plasticity (STDP), where, if a presynaptic neuron fires a few milliseconds before a post-synaptic neuron, the strength of the synapse is increased. Conversely, if the presynaptic neuron fires after the post-synaptic neuron, the strength of the synapse is diminished (27). That is, the timing issue is crucial for outcome. In an effort to circumvent the vulnerability of this time-dependency, Tolmacheva et al. (24) have suggested a protocol consisting of high-intensity, 0.2 Hz TMS paired with short 100 Hz bursts (instead of one isolated pulse) of PNS (so-called “high-PAS”).

Previous studies using this protocol have been suggested to yield improved motor function (24, 28). However, these preliminary results call for corroboration by further studies from other centres. The present study thus purports to add to the evidence base for the efficacy or otherwise of this protocol in improving upper extremity motor function in chronic-stage motor incomplete SCI.

## METHODS

### Inclusion criteria

*All of*: acquired chronic sensorimotor incomplete (AIS C or D) SCI with significant motor impairment of functional relevance in the presence of some residual motor function (grade 1–4) in at least 2 of 5 key muscles in the upper extremity; NLI C7 or rostral; resident of the county comprising the catchment area of the Department of Rehabilitation Medicine, Linköping University Hospital (Region Östergötland, Sweden); time since injury >1.5 years.

### Exclusion criteria

*Any of:* severe comorbidity (such as terminal cancer, psychosis, or other diseases likely precluding full participation); age >80 years; established contraindications to TMS; inability to communicate in Swedish or English.

### Participants

Participants were identified from registries at the Department of Rehabilitation Medicine, Linköping University Hospital. Medical records were screened for eligibility. Individuals fulfilling the study criteria were contacted by the first author (CW) and invited to participate. Five subjects fulfilling the criteria gave written informed consent and participated in the study. The study was approved by the Swedish Ethical Review Authority prior to recruitment. All experiments were performed in accordance with the Declaration of Helsinki. Data were collected between September of 2019 and August of 2023.

### Clinical evaluation

The assessment protocol was administered 3 times for each participant: t_0_ = baseline (the *week immediately preceding* the stimulation period), t_1_ = post-stimulation (the *week immediately after* the stimulation period), and t_2_ = the *1-month follow-up*, 4–5 weeks after the stimulation period had finished. The assessment protocol was performed jointly by 2 therapists, blinded to stimulation parameters (including target extremity).

The assessment protocol (Appendix S1) comprised:

- Manual muscle testing (MMT) using a scale from 0–5 according to Daniel and Worthingham (29), and a standardized grip strength measurement using a JAMAR device (30), where the average value of 3 measurements for each hand at each assessment was determined.
- Each participant was asked to formulate 1 to 3 individualized treatment goals, which were assessed using the Canadian Occupational Performance Measure (COPM (31)). If more than 1 treatment goal was chosen, the average values for performance and satisfaction for these goals were used for the purpose of statistical comparisons.
- As studies have indicated that neuromodulatory interventions may alleviate (32, 33) or exacerbate (34) pain, patients were also asked if any pain was present during the last 24 h, and, if so, to describe its character, distribution, and intensity using a numeric rating scale (NRS) ranging from 0–10. Participants were asked to continue with unaltered pain medication, if any, for the duration of the study.

The *primary endpoint* was set as the average change in MMT scores from the baseline to the post-stimulation assessment (MMT(t_1_ –t_0)_) in the targeted extremity. (“Target” referring to the extremity subjected to PAS, and “off-target” to the contralateral side, cf. below.) *Secondary endpoints* were changes in grip strength, performance (COPM-P_(_t_1_ –t_0)_), satisfaction (COPM-S_(_t_1_ –t_0)_), pain intensity and the average change in MMT scores from the baseline to the post-stimulation assessment (MMT_(_t_1_ – t_0)_) in the *off-target* extremity. Additionally, comparisons were made between baseline values and the 1-month follow-up (i.e., t_2_ – t_0_) to see whether improvements, if any, were sustained.

### Paired-associative stimulation

The method employed in this study was chosen to correspond to that previously described (25): a structural T1-weighted brain MRI scan was imported into the Nexstim eXimia machine (Nexstim, Helsinki, Finland) in order to enable highly precise repetitive navigated transcranial magnetic stimulation (rnTMS). Using the Nexstim Navigated Brain Stimulation 5.2.4 software, stimulation hot spots in the motor cortex contralateral to the targeted extremity were determined for m. abductor digiti minimii (ADM; n. ulnaris), m. abductor pollicis brevis (APB; n. medianus), and m. extensor digitorum communis (EDC; n. radialis). The weaker hand was targeted for stimulation in all patients.

Motor evoked potentials (MEPs) were obtained using nTMS over the primary motor cortex. Simultaneous EMG recording (Nicolet Biomedical, EMG surface electrode, Cephalon A/S, Nørresundby, Denmark) was made from the respective index muscle (cf. above). Each of the 3 above-mentioned hot spots was defined as the MRI coordinate with the lowest resting motor thresholds (RMT) for the respective key muscle. As a measure of local cortical excitability, RMTs were determined for each motor hot spot, defined as the field strength (V/m) required to elicit MEPs of at least 50µV, at least 50% of the time. This indicates the optimal stimulation hot spots (i.e., with the lowest RMT for the respective key muscle). The PAS paradigm relies on the principle of STDP, as described in the introduction. Therefore, to optimize timing of the 2 stimulation modalities relative to each other, the average MEP latency (aMEPL) obtained during stimulation at 120% of RMT was noted for each hot spot.

Nerve conduction studies were performed for the ulnar, median, and radial nerves using a Dantec Keypoint electroneurography machine (Natus Neurology, Middleton, WI, USA). A hand-held bipolar stimulating electrode (Natus Neurology, Middleton, WI, USA) was used for stimulation and surface electrodes (Neuroline 720, AMBU A/S, Ballerup, Denmark) for recording as previously described (25). This yields 2 kinds of data necessary for PAS (25), for each nerve: (*i*) the stimulus intensity required to elicit an F-response using a 1 ms pulse (“PNS intensity”, given in mA) and (*ii*) the shortest F-response latency using supramaximal stimulation with a 0.2 ms pulse (“PNS latency”, given in ms). The F-response (or F wave) is a late motor response seen after supramaximal electrical stimulation of a peripheral motor nerve, and represents a kind of “echo” from the spinal cord segment that gives rise to the stimulated motor nerve (35). To facilitate the following PAS sessions, the exact spots for electrode placement were indicated on the patient’s skin using a semi-permanent marker.

The PAS comprised rnTMS and PNS given synchronously for 20 min for each of the 3 cortical hot spot/nerve pairs. The rationale behind targeting all 3 pairs was to achieve optimal improvements in motor function. PAS was administered according to Tolmachena et al. (24), using the following parameters, determined individually for each participant, at the first stimulation session: 1 TMS pulse was administered every 5 s at 100% of maximum stimulator output. Concurrent with each TMS pulse, a 50 ms, 100 Hz train of PNS was given at “PNS intensity” (defined above). To achieve synchronization at the corticospinal–motoneuronal synapse, stimulation triggers were offset by the differential of the respective latencies, irrespective of which of them were longest (i.e., aMEPL – PNS latency or PNS latency – aMEPL).

For each participant, 16 sessions of PAS were administered over 4 weeks: 5 sessions per week for the first 2 weeks, then 3 sessions per week for the following 2 weeks. After 2 weeks (10 sessions), PNS intensity, PNS latency, and aMEPL were measured again, and the stimulation parameters were updated accordingly. TMS stimulation parameters were not altered. For PNS, minor adjustments regarding stimulation intensity (in the order of 10–20%) were made for 2 patients. This was done to reduce discomfort.

### Statistics

Analyses were performed using IBM SPSS v. 27 (IBM Corp, Armonk, NY, USA). Data are presented as means and standard deviations for normally distributed continuous variables (assessed using Shapiro–Wilks tests); as medians and ranges for non-normally distributed numeric variables; and as *n* (%) for categorical data. Comparisons over time for ordinal data were made using paired Wilcoxon signed-rank tests. Comparisons for normally distributed continuous variables were made using *t*-tests. No imputation was performed. *P*-values < 0.05 were considered statistically significant unless otherwise noted.

## RESULTS

Basic descriptors of study participants are given in Table I.

**Table I:** Basic descriptive data

ID	Age	Hand	Sex	Mechanism	NLI	AIS	SCI duration (years)	Comorbidities	Medication
1	64	L	F	Tumour	C2	D	11	ET, DM, HF, O	PRE 600
2	58	R	M	MVA	C2	D	30	Whiplash	PRE 300
3	63	R	F	Degen.	C5	D	5	Arnold-Chiari	None
4	73	R	F	Fall	C2	D	4	HT	BAC 30
5	62	R	F	Degen.	C3	D	2	RA, Uln entrap (Con)	BAC 10, GAB 1800

MVA: motor vehicle accident; Degen: degenerative myelopathy; NLI: neurological level of injury; AIS: ASIA Impairment Scale; NP: neuropathic pain; PRE: pregabalin; BAC: baclofen; GAB: gabapentin; CNS-active medications (for spasticity and/or neuropathic pain) are shown as milligrams per day; Hand: hand dominance; L=left; R=right; ET=essential tremor, DM=diabetes mellitus, HF=heart failure; O=obesity; HT=hypertension; RA=rheumatoid arthritis, Uln entrap (Con)=entrapment of the ulnar nerve (contralateral extremity).

All patients had incomplete cervical SCI, NLI C2–C5, all AIS D. The mechanism of injury was traumatic (3/5) or degenerative (2/5). Four out of five were female. Time since injury varied between 2 and 30 years. Four out of five were treated with medication for spasticity and/or neuropathic pain.

The group values regarding primary and secondary outcomes are presented in Tables II–VI.

**Table II:** Primary outcomes: group values of average muscle function scores over time

Arm	MMTt_1_-t_0_	*p*-value	T/Off-T immediate	MMTt_2_-t_0_	p	T/Off-T follow-up
Target	+0.49	0.078	1.31	+0.55	0.062	1.14
Off-target	+0.37	0.026		+0.49	0.040	

Muscles with grade 5/5 at the baseline assessment are excluded from analysis. “Target” refers to the extremity targeted with PAS, and “off-target” to the contralateral side. “T/Off-T immediate” is the ratio of the improvement in MMT score from *baseline to t*_1_ in the *stimulated* extremity divided by the corresponding score in the *non-stimulated* extremity. Similarly, “T/Off-T follow-up” is the corresponding ratio of the improvement in MMT score from *baseline to t*_2_.

MMT: manual muscle testing; t_0_: baseline; t_1_: post-stimulation follow-up; t_2_: 1-month follow-up.

**Table III:** Secondary outcomes: group values of individualized goals assessed on the Canadian Occupational Performance Measure (COPM)

Subscale	COPMt_0_	COPMt_1_	COPMt_2_	COPMt_1_-t_0_	*p*-value	COPMt_2_-t_0_	*p*-value
Performance	3.6	5.7	5.6	+2.1	0.10	+2.0	0.14
Satisfaction	3.1	5.5	5.0	+2.4	0.72	+1.9	0.29

Performance and Satisfaction are graded independently using a numeric rating scale from 0–10. If a patient had multiple goals, mean scores are shown for each time point.

t_0_: baseline; t_1_: post-stimulation follow-up; t_2_: 1-month follow-up.

**Table IV:** Secondary outcomes: group values of grip strength

Group	Hand	Forcet_0_	Forcet_1_	Forcet_2_	Forcet_1_-t_0_	*p*-value	Forcet_2_-t_0_	*p*-value
PAS (*n* = 5)	Target	13.2	16.4	16.6	+3.2	0.14	+3.4	0.23
	Off-target	20.3	22.5	23.9	+2.2	0.04	+3.6	0.14

Grip strength over time, displayed in kg. At each time point, 3 measurements of maximum voluntary hand grip strength were averaged from each hand individually.

t_0_: baseline; t_1_: post-stimulation follow-up; t_2_: 1-month follow-up. “Target” refers to the extremity targeted with PAS, and “off-target” to the contralateral side.

**Table V:** Secondary outcomes: group values of pain intensity

Group	Hand	NRSt_0_	NRSt_1_	NRSt_2_	NRSt_1_-t_0_	*p*-value	NRSt_2_-t_0_	*p*-value
PAS (*n* = 5)	Target	4.5	3.5	4.4	–1	0.28	–0.1	0.18
	Off-target	3.3	2.8	3	–0.5	0.58	–0.3	1.00

Pain intensity assessed using a numeric rating scale (NRS) from 0–10, recalled for the last 24 h at each clinical evaluation.

t_0_: baseline; t_0_: post-stimulation follow-up; t_2_: 1-month follow-up. “Target” refers to the extremity targeted with PAS, and “off-target” to the contralateral side. _Averages are given for the PAS group._

**Table VI:** Secondary outcomes: individual scores of individualized goals assessed on the Canadian Occupational Performance Measure (COPM)

Pat #	Goal	COPMt_0_	COPMt_1_	COPMt_2_
1	Apply brakes on walker with right hand	P: 1 S: 1	P: 1 S: 1	P: 1 S: 1
1	Cut bread	P: 5 S: 3	P: 4 S: 4	P: 3 S: 2
2	Work out with both hands simultaneously (push-up machine)	P: 6 S: 5	P: – S: –	P: – S: –
3	Work on a workbench with normal height	P: 5 S: 5	P: 7 S: 7	P: 7 S: 9
3	Perform one push-up on the knees	P: 5 S: 5	P: 8 S: 10	P: 6 S: 8
3	Normal arm swing while walking	P: 5 S: 5	P: 7 S: 7	P: 8 S: 9
4	Button trousers independently	P: 1 S: 1	P: 8 S: 10	P: 8 S: 8
4	Pull up trousers independently	P: 6 S: 5	P: 7 S: 7	P: 6 S: 6
4	Use a kitchen knife with the weaker hand	P: 4 S: 4	P: 9 S: 10	P: 7 S: 7
5	Eat with cutlery using the right hand	P: 1 S: 1	P: 4 S: 1	P: 4 S: 1
5	Write (solve crossword puzzle, write shopping list)	P: 3 S: 2	P: 4 S: 2	P: 6 S: 2
5	Use cell phone independently	P: 4 S: 2	P: – S: –	P: 6 S: 2

t_0_: baseline; t_1_: post-stimulation follow-up; t_2_: 1-month follow-up; P: Performance; S: Satisfaction.

At the group level, MMT scores increased modestly in both extremities, with a slightly greater improvement at the follow-up, compared with post-stimulation, with varying results at the individual level. Similarly, COPM scores increased modestly at the group level with large variations between individuals. Grip strength increased in both extremities. Pain intensity was unchanged.

**Fig. 1:**
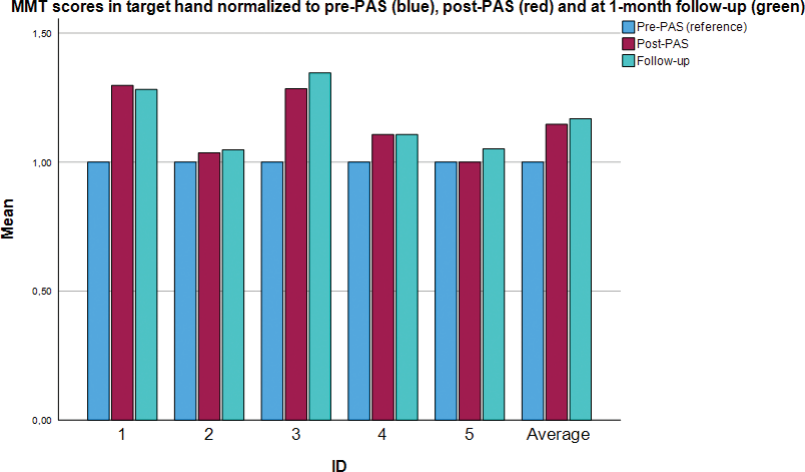
Individual and group values: normalized manual muscle testing scores (0–5) of the *targeted* upper extremity. PAS: paired associative stimulation. Values are normalized using the pre-PAS measurement (blue) as a baseline for comparison. The red bar denotes the post-PAS measurement and the green bar denotes the final measurement (at the 1-month follow-up). Muscles with an MMT score of 5 at the pre-PAS measurement are excluded from analysis. The figure shows individual values as well as the group mean (rightmost bar cluster).

Fig. 1 shows individual and group-level data of MMT scores for the targeted extremity. At the group level modest improvements were seen, albeit just outside statistical significance (*p* = 0.06). At the individual level, 2 patients responded well in the targeted extremity, 1 responded modestly and 2 did not.

**Fig. 2:**
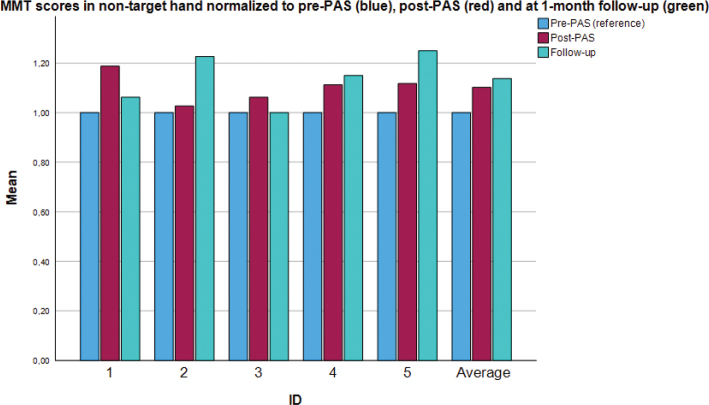
Individual and group values: normalized manual muscle testing scores (0–5) of the *non-targeted* upper extremity. PAS: paired associative stimulation. Values are normalized using the pre-PAS measurement (blue) as a baseline for comparison. The red bar denotes the post-PAS measurement and the green bar denotes the final measurement (at the 1-month follow-up). Muscles with an MMT score of 5 at the pre-PAS measurement are excluded from analysis. The figure shows individual values as well as the group mean (rightmost bar cluster).

Fig. 2 shows individual and group-level data of MMT scores for the non-targeted extremity. A statistically significant effect was seen at the group level (*p* = 0.04), suggesting improvement in motor function.

Compared with the results for the targeted extremity, those of the non-targeted extremity were more variable. All participants but 1 showed some improvement, with variable timing.

**Fig. 3:**
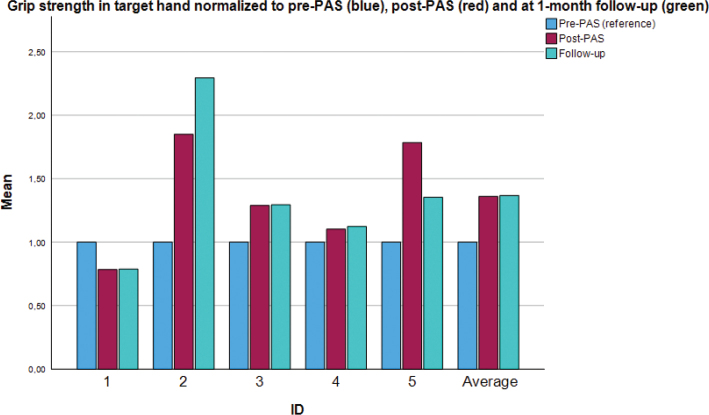
Individual and group values: normalized grip strength force of the *targeted* upper extremity. PAS: paired associative stimulation. Values are normalized using the pre-PAS measurement (blue) as a baseline for comparison. The red bar denotes the post-PAS measurement and the green bar denotes the final measurement (at the 1-month follow-up). The figure shows individual values as well as the group mean (rightmost bar cluster).

Fig. 3 shows individual and group-level data of grip strength for the targeted extremity. At the group level, there was an increase in grip strength of approximately 40% in the targeted extremity, sustained at the follow-up (see also Table IV). However, this was only close to being statistically significant (*p* = 0.14 and *p* = 0.23, respectively). At the individual level, 1 participant showed a major increase in grip strength (almost double post-stimulation and more than double at the follow-up). One participant’s grip strength increased by approximately 75% post-stimulation, but this was only partially sustained at the 1-month follow-up. The remaining 3 participants showed small improvements (10–30%) in grip strength, or even a slight decrease.

**Fig. 4:**
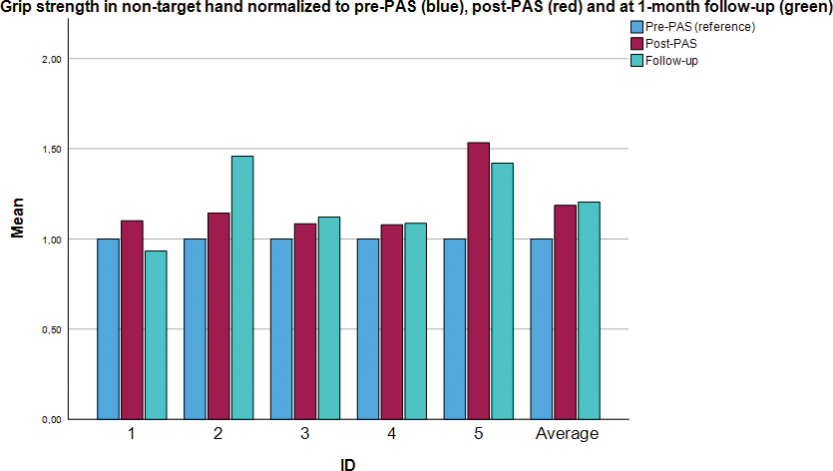
Individual and group values: normalized grip strength force of the *non-targeted* upper extremity. PAS: paired associative stimulation. Values are normalized using the pre-PAS measurement (blue) as a baseline for comparison. The red bar denotes the post-PAS measurement and the green bar denotes the final measurement (at the 1-month follow-up). The figure shows individual values as well as the group mean (rightmost bar cluster).

Fig. 4 shows individual and group-level data of grip strength for the non-targeted extremity. Interestingly, the improvement in the non-targeted extremity, albeit apparently smaller, did reach statistical significance initially (*p* = 0.04 and *p* = 0.14, see also Table IV). At the individual level, 1 participant showed a moderate increase in grip strength at the post-stimulation measurement (t_1_), and 2 did so at follow-up (t_2_). For the remaining 3 participants, grip strength was virtually unchanged for the duration of the study.

Performance on the COPM increased at the group level (Table III) by 2.1 points post-stimulation and by 2.0 points at the 1-month follow-up. Similarly, Satisfaction increased by 2.4 and 1.9 points, respectively. At the individual level, clear differences were seen (Table VI).

Some goals were not close to being reached; others were well so. Patient 1 deteriorated slightly. Patient 2 did not attempt his goal during the stimulation period. Patient 3 experienced significant improvements regarding all 3 specified goals (“Work on a workbench with normal height”, “Perform one push-up on the knees”, and “Achieve normal arm swing while walking”). Patient 4 improved regarding 2 out of 3 goals (“Button trousers independently” and “Use a kitchen knife with the weaker hand”, but not “Pull up trousers independently”). Patient 5 improved modestly regarding Performance but not Satisfaction (“Eat with cutlery using the right hand”).

### Subjective sensations/adverse events/other comments

No serious adverse events were observed or reported. However, most participants experienced light to moderate pain from PNS, especially during the first few sessions. Lidocaine/prilocaine cream (EMLA, 25 mg/g / 25 mg/g) was offered. Minor discomfort was reported from the noise and/or skin sensation induced by TMS. None abstained from stimulation due to experience of pain or for any other reason. There was no indication of aggravation of pain outside of the stimulation itself. Rather, as shown in Table V, there was a trend for decreased pain intensity after the stimulation period.

All participants attended all 16 PAS sessions, except for 1 participant (who missed 1 session) and all planned clinical assessments.

## DISCUSSION

Improvements, although not reaching statistical significance at the group level, were found in MMT scores for the targeted extremity. MMT increased by on average 0.5 points during the stimulation period, sustained at the 1-month follow-up. At the individual level, the results were quite varied. Two participants show quite clear improvements in MMT scores in the targeted extremity, which was sustained at the 1-month follow-up. For the remaining 3 participants, the results were less clear. One possible explanation for the apparent “volatility” of the results seen in the non-targeted extremity may be due to the fact that it typically had fewer muscles eligible for analysis, as more muscles were rated 5/5 at the initial assessment.

We also observed moderate increases in grip strength during the stimulation period, which remained at the 1-month follow-up. For most individualized goals assessed using the COPM, clinically, albeit not statistically significant, improvements were seen. A change of 2 points on either subscale is usually considered clinically significant. In this study, results were heterogeneous: the change in COPM scores ranged from slight deterioration to major improvements. At the group level, the mean improvement was about 2 points for both Performance and Satisfaction. Thus, PAS as here administered over a short period, enabled persons with chronic tetraplegia to reach their improvement goals.

Although in agreement with earlier studies (23, 24, 28), the effects reported here were more modest. In our study, average time since injury was 10 years as compared with 4 years (28) and 7 years (24) in previous studies. As the plasticity of the spinal circuitry is believed to decrease with time (36, 37), longer time since injury may impair the therapeutic response. It should also be noted that the stimulation period was longer (6 weeks) in 1 of these studies (24). Furthermore, our participants had a mean age of 64 years, as compared with 48 years (28) and 44.2 years (26) in previous studies. Again, this age difference may have had an influence, as it has been proposed that the plasticity of the central nervous system decreases with age (38–40). Finally, it has been described (33, 34) that the PAS effect may be negatively influenced by concurrent medication against spasticity and/or neuropathic pain, such as pregabalin, gabapentin, and baclofen. In the current study, however, participants were not asked to discontinue any medication. It is generally assumed that the effects of rehabilitation, including neuromodulation, are larger early as compared with late after an injury. It is therefore hopeful that the patient (No. 2) with the longest post-injury time (30 years), demonstrated impressive increases in hand function after stimulation.

Interestingly, MMT scores for the *non-targeted* extremity also improved, although on average less so than for the targeted arm. The same pattern has been observed in earlier, similar studies (24, 28). It has been shown that the balance between excitation and inhibition is altered in the CNS after SCI, both at the spinal level (41) and at the cortical level (42). One hypothesized mechanism of action for PAS is normalization of this balance. It has been suggested that this can occur bilaterally even if only one side is deliberately targeted (43, 44). To the best of our knowledge, no study has explored whether this is indeed the underlying mechanism. Alternatively, improved motor function in 1 limb may additionally inspire increased use of the contralateral limb.

It is also in principle possible that the TMS at maximal stimulator output also activated parts of the motor cortex contralateral to the intended target. Even the peripheral nerve stimulation could also, speculatively, activate the neural circuitry of the non-targeted arm through aberrant nerve connections induced by the injury (25). It is possible that the larger improvement of the non-targeted hand may be attributed to it being less severely affected. The minor changes of PNS stimulation intensity made in 2 patients in all probability did not affect the outcome for these individuals. We could find no traits identifying in which patients such changes were necessary. Medical records were screened with regard to frequency of rehabilitative interventions and we see no indication of the impairment of rehabilitative interventions for the individuals in this study caused by the pandemic.

### Limitations

One obvious limitation was the small number of participants in the study. Inclusion criteria werenarrowly specified as regards degree of extant residual motor function. It was postulated that subjects with some residual motor function albeit of no, or minimal, practical usefulness were those most likely to respond to stimulation and achieve clinically useful gains thereof. Participation criteria were also set so as to exclude recently injured patients, in order to avoid inevitable bias imposed by the expected spontaneous functional improvement, as well as improvements due to simultaneously occurring routine multimodal neurorehabilitative interventions provided in that phase. Our aim was thus to study effects of stimulation at a chronic stage where the impact of other factors is minimal, something which restricted an already small recruitment base. However, as the motive for “proof of concept” is still a high priority, we believe this choice of restrictiveness in inclusion can be justified.

Second, and for purely practical reasons, we had no possibility to offer participants inpatient beds for the duration of the study. Thus, participants had to be able to commute to and from the hospital on an almost daily basis, thus further restricting the number of persons willing and/or able to participate in the study.

### Conclusion

This study corroborates previous studies showing modest improvements in upper extremity motor function by a 4 week/16 session paired associative stimulation programme, sustained at 1 month follow-up post stimulation. It extends our knowledge by demonstrating the capacity of this neuromodulatory technique to improve function in, compared with earlier studies, older patients at a later time after injury. Larger future studies will clarify the optimal timing and patient characteristics for this type of treatment.

## Supplementary Material

PAIRED ASSOCIATIVE STIMULATION IMPROVES MOTOR FUNCTION IN THE UPPER EXTREMITY IN CHRONIC INCOMPLETE SPINAL CORD INJURY: A CORROBORATIVE STUDY
