# Supplementary material for: PAIRED ASSOCIATIVE STIMULATION IMPROVES MOTOR FUNCTION IN THE UPPER EXTREMITY IN CHRONIC INCOMPLETE SPINAL CORD INJURY: A CORROBORATIVE STUDY
Source: J Rehabil Med. 2024 Nov 13;56:41021. doi: 10.2340/jrm.v56.41021 (PMC11579534; doi:10.2340/jrm.v56.41021)
Supplement: PAIRED ASSOCIATIVE STIMULATION IMPROVES MOTOR FUNCTION IN THE UPPER EXTREMITY IN CHRONIC INCOMPLETE SPINAL CORD INJURY: A CORROBORATIVE STUDY [file JRM-56-41021-s1.pdf]

Supplementary material has been published as submitted. It has not been copyedited, or typeset by Journal of Rehabilitation Medicine

Appendix S1

Participant initials:

Time point: ☐Before stimulation ☐ After stimulation ☐ 1-month follow-up

Date:

Testers (Circle): VJ LO SA EH Other, specify:

Dominant hand: ☐ Right ☐ Left

Specify at least one personalized goal to be evaluated after stimulation.

Goal 1 (graded according to COPM):

Performance (1-10):

Satisfaction (1-10):

Goal 2 (graded according to COPM):

Performance (1-10):

Satisfaction (1-10):

Goal 3 (graded according to COPM):

Performance (1-10):

Satisfaction (1-10):

Pain arm/hand, last 24h? ☐ Yes ☐ No

If present, which side(s)? ☐ Left ☐ Right ☐ Both

Describe the character of the pain:

Localisation:

Rate the average pain intensity over the last 24 hours (0 is no pain and 10 is the worst imaginable pain)

NRS 0-10 left:

NRS 0-10 right:

Manual muscle testing (MMT) (0-5) – Start with the dominant hand

The strength in each muscle specified below is tested using the instructions in the book and graded from 0-5 (MMT, Daniel and Worthingham), where 0 = total paralysis; 1 = palpable or visible contraction; 2 = active motion, full ROM with gravity removed; 3 = full ROM against gravity; 4 = full ROM against moderate resistance in a muscle specific position; 5 = normal strength.

NT = Not tested, Please comment why.

Muscle (nerve)	Side of body			
brachioradialis (n. rad)	Right:___	☐NT, comm:_____	Left:___	☐Not tested
biceps brachii (armb.flex, (n. rad)	Right:___	☐NT, comm:_____	Left:___	☐Not tested
pronator teres + quadratus (N. med):	Right:___	☐NT, comm:_____	Left:___	☐Not tested
supinator and b. brachii (sup.) (n. rad)	Right:___	☐NT, comm:_____	Left:___	☐Not tested
triceps brachii (n. rad)	Right:___	☐NT, comm:_____	Left:___	☐Not tested
extensor carpi radialis (n. rad)	Right:___	☐NT, comm:_____	Left:___	☐Not tested
extensor carpi ulnaris (n. rad)	Right:___	☐NT, comm:_____	Left:___	☐Not tested
flexor carpi radialis (N. med)	Right:___	☐NT, comm:_____	Left:___	☐Not tested
flexor carpi ulnaris (n. uln)	Right:___	☐NT, comm:_____	Left:___	☐Not tested
dorsal interossei (n. uln)	Right:___	☐NT, comm:_____	Left:___	☐Not tested
abductor digiti minimi (n. uln)	Right:___	☐NT, comm:_____	Left:___	☐Not tested
palmar interossei (n. uln)	Right:___	☐NT, comm:_____	Left:___	☐Not tested
flexor digitorum superficialis (N. med)	Right:___	☐NT, comm:_____	Left:___	☐Not tested
flexor dig. prof. (dig. II-III) (N. med)	Right:___	☐NT, comm:_____	Left:___	☐Not tested
flexor dig. Prof. (dig. IV-V) (n. uln)	Right:___	☐NT, comm:_____	Left:___	☐Not tested
extensor digitorum (n. rad)	Right:___	☐NT, comm:_____	Left:___	☐Not tested
flexor pollicis brevis (N. med)	Right:___	☐NT, comm:_____	Left:___	☐Not tested
flexor pollicis longus (N. med)	Right:___	☐NT, comm:_____	Left:___	☐Not tested
opponens pollicis (N. med)	Right:___	☐NT, comm:_____	Left:___	☐Not tested
adductor pollicis (n. uln)	Right:___	☐NT, comm:_____	Left:___	☐Not tested
abd. poll. long. (n.rad) + brev. (n. med)	Right:___	☐NT, comm:_____	Left:___	☐Not tested
extensor pollicis longus (n. rad)	Right:___	☐NT, comm:_____	Left:___	☐Not tested
extensor pollicis brevis (n. rad)	Right:___	☐NT, comm:_____	Left:___	☐Not tested

Grip strength dynamometry (JAMAR, average of three trials):

Left hand:

Right hand:
